# Spatiotemporal Dynamics of Microcystin Variants and Relationships with Environmental Parameters in Lake Taihu, China

**DOI:** 10.3390/toxins7083224

**Published:** 2015-08-18

**Authors:** Xiaomei Su, Qingju Xue, Alan D. Steinman, Yanyan Zhao, Liqiang Xie

**Affiliations:** 1 State Key Laboratory of Lake Science and Environment, Nanjing Institute of Geography and Limnology, Chinese Academy of Sciences, 73 East Beijing Road, Nanjing 210008, China; E-Mails: suxiaomei_2008@163.com (X.S.); qingju871228@163.com (Q.X.); yyzhao@niglas.ac.cn (Y.Z.); 2 College of Resources and Environment, University of Chinese Academy of Sciences, Beijing 100049, China; 3 Annis Water Resources Institute, Grand Valley State University, 740 West Shoreline Drive, Muskegon, MI 49441, USA; E-Mail: steinmaa@gvsu.edu

**Keywords:** microcystins, cyanobacteria, distribution, environmental parameters, Lake Taihu

## Abstract

Excessive anthropogenically-caused nutrient loading from both external and internal sources has promoted the growth of cyanobacteria in Lake Taihu from 2005 to 2014, suggesting increased production and release of cyanotoxins. In order to explain the spatial distribution and temporal variation of microcystins (MCs), the intracellular concentrations of MCs (MC-LR, -RR and -YR, L, R and Y are abbreviations of leucine, arginine and tyrosine) were monitored monthly from July 2013 to June 2014. Three MC variants are present simultaneously in Lake Taihu; the MC-LR and -RR variants were dominant (accounting for 40% and 39% of the total), followed by MC-YR (21%). However, MC-YR accounted for a higher proportion in colder months, especially in March. The highest concentrations of intracellular MCs were found in July and October when cyanobacteria cell density also reached the maximum. The average concentrations of MC-LR, -RR and -YR in July were 4.69, 4.23 and 2.01 μg/L, respectively. In terms of the entire lake, toxin concentrations in northern parts were significantly higher than the eastern part in summer, when MC concentrations were several times higher than the guideline value by WHO throughout much of Lake Taihu. Results from correlation and redundancy analysis (RDA) showed that total MCs, including all variants, were strongly and positively correlated with cyanobacteria cell density, water temperature, total phosphorus (TP) and pH, whereas each variant had different correlation coefficients with each of the considered environmental variables. MC-RR showed a stronger relationship with temperature, in contrast to MC-YR and -LR. Dissolved inorganic carbon (DIC) showed a negative relationship with each variant, suggesting that rising DIC concentrations may inhibit cyanobacterial growth and thereby reduce MC production in the future.

## 1. Introduction

The frequent occurrence of cyanobacterial harmful algal blooms (CHABs) poses a serious threat to animal and human health because of their ability to produce a wide variety of toxins throughout the world [1,2]. Climate change and global warming may increase the frequency and density of cyanobacterial blooms in freshwater systems [3,4]. Cyanotoxins, secondary metabolites produced by intensive CHABs, can threaten the supply of drinking water and fisheries-related food supplies [5,6]. In addition, the toxins can accumulate in organisms and be transferred via aquatic food webs, presenting potential risks to human health [7].

Microcystins (MCs) are considered to be the most widely-distributed and dangerous hepatotoxic compounds compared to other cyanotoxins, such as nodularins and cylindrospermopsin [8]. MCs are potent inhibitors of protein phosphatases 1 and 2A for both plants and animals and are produced predominantly by freshwater genera, including *Microcystis*, *Planktothrix* and *Anabaena* [9]. The incident that happened at a hemodialysis center in Caruaru, Brazil, in 1996 proved that MC-contaminated water can lead to hepatic disease or death [10]. The development of rapid, inexpensive and sensitive monitoring methods [11] has resulted in the discovery and detection of an increasing diversity of MCs in lakes [12,13,14,15,16,17], rivers [18], reservoirs [19,20,21] and ponds [22] all over the word, suggesting that MC pollution has become a global scientific problem in recent years. To date, more than 90 variants of MCs with variable toxicity have been identified from cyanobacterial blooms and cultures [8]. Among them, MC-LR, -RR and -YR (L, R and Y are abbreviations of leucine, arginine and tyrosine) are the most commonly reported in natural waters. Because of varying levels of toxicity for each MC variant [23], the proportion of dominant MC variants will have an important influence on overall toxicity. In terms of the three common MC variants, toxicological studies on mice have shown that MC-LR is the most toxic, followed by MC-YR and -RR, according to LD_50_ values. However, MCs were typically reported as a total concentration, with relatively few studies evaluating the effect of environmental factors on the MC variants and their relative abundances in the freshwater systems [24].

The production of MCs is affected not only by cyanobacteria biomass directly, but also by environmental factors indirectly [25]. A series of studies on the relationship between total MC production and environmental factors has been conducted in many freshwater lakes [22,26,27,28,29], but these results were not consistent with each other, and few of them have examined the distribution of different MC variants and their percentages among lakes. In Lake Vancouver, PO_4_–P was found to be the main factor influencing intracellular concentrations of MCs [12]. Water temperature was confirmed to be positively correlated with concentrations of MCs in the Daechung reservoir of Korea [28]. Higher concentrations of MCs were attributed to the high biomass of cyanobacteria coupled with the lake’s eutrophication status, as indicated by high Chl-a content, high nutrient load and low dissolved oxygen (DO) in two fresh water ponds in India [22]. Higher temperatures (>25 °C) have been shown to enhance MC-RR production, whereas lower temperatures favored MC-LR synthesis [30]; different nitrogen forms influenced concentrations of MCs and the composition via changes in cyanobacterial community structure based on a survey of three lakes in Canada [24], and the ratio of MC variants changed in response to differing light intensities [31]. However, which environmental parameter is crucial to affect the cyanobacterial community and its ability to produce different variants is still unresolved. This issue has both ecological and societal relevance, as understanding the transformation of MC-LR to the less toxic MC-YR or -RR variants may result in management strategies to reduce toxin production. Therefore, it is important to identify not only the key factors influencing total concentration of MCs, but also to understand which factors are responsible for regulating the composition of MC variants.

Lake Taihu is the third largest freshwater lake in China with an area of more than 2000 km^2^. As a shallow lake with a mean depth of less than 2 m, excessive anthropogenic nutrient loading from both external and internal sources has contributed to its serious eutrophication. Previous studies on the distribution of MCs were concentrated in Meiliang Bay [32,33,34,35], Zhushan Bay [36] or other bays [37] and some sites located in the lake’s eastern part [38]. However, these results do not reflect MC production and distribution in the entire lake over temporal and spatial scales, and no studies have examined the relationship of the distribution of MC variants with the environmental parameters among the entire lake. Furthermore, it is important to study MCs beyond just the summer, as cyanobacteria are still abundant in autumn, and they will release a large amount of MCs during post-bloom periods [37].

The main objectives of this research are to describe the long-term dynamics of changes in cyanobacteria cell density in Lake Taihu from 2005 to 2014, in addition to spatial and temporal variations of intracellular MCs, including MC-LR, -RR and -YR of the entire lake from 2013 to 2014. Furthermore, this study examined the relationship between environmental variables, alone or in combination with each other, and cyanobacteria abundance, total concentrations of MCs and the composition of MC variants.

## 2. Results

### 2.1. Dynamic Changes of Cyanobacteria Cell Density from 2005 to 2014

**Figure 1:**
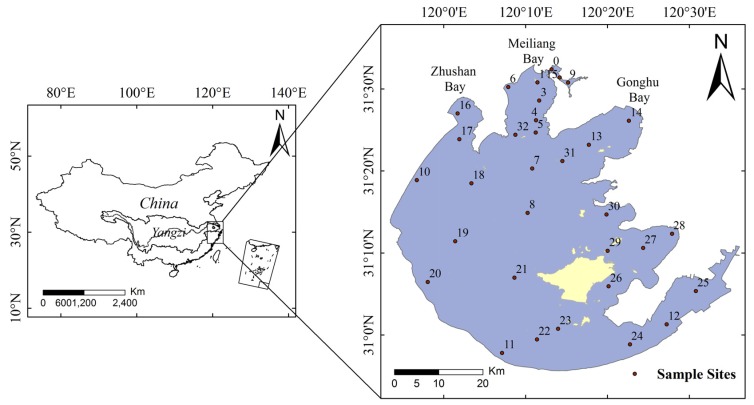
Location of Lake Taihu in China (inset) and sampling sites (0–32) in this study.

A total of 32 sampling sites were covered in this study. Among them, 14 sites (0, 1, 3, 4, 5, 6, 7, 8, 10, 13, 14, 16, 17, 32) were located in the northern part of Lake Taihu (Figure 1).

**Figure 2:**
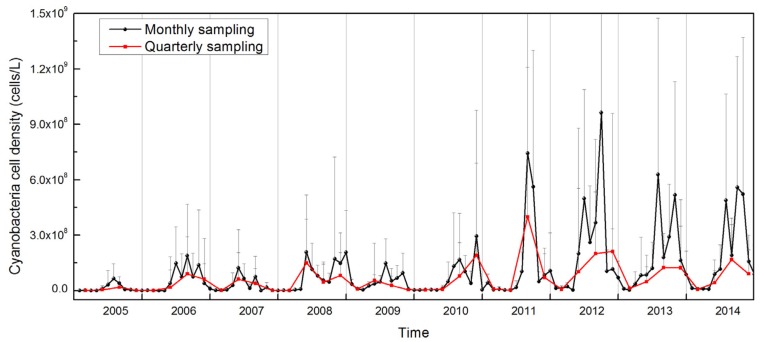
The variability of cyanobacteria cell density under two sampling frequencies dating from 2005 to 2014 in Lake Taihu. Monthly sampling focused on the most polluted areas; quarterly sampling included sites throughout the entire lake. Error bars represent the standard deviation.

Monthly sampling was conducted in the heavily-polluted areas, while quarterly sampling was conducted in the entire lake with 32 sampling sites (Figure 2). Cyanobacteria cell density during the past ten years exhibited distinct temporal and spatial trends. The greatest abundance under monthly sampling was in October 2012, with values of approximately 1 × 10^9^ cells/L, while the maximal abundance under quarterly sampling was in August 2011, with a value of 4 × 10^8^ cells/L (Figure 2).

The monthly abundance of cyanobacteria remained at low levels with less than 3 × 10^8^ cells/L, until 2011, when densities began to increase rapidly and then stayed at high levels through 2014. The highest values of cyanobacteria cell density based on quarterly sampling also exhibited a slight increase over the past five years. Cyanobacteria cell density estimates using these two sampling regimes were close to each other prior to 2011, after which they began to diverge and remained that way through 2014. Cyanobacteria were much more abundant in the northern parts of Lake Taihu than the other lake areas, and this was more pronounced during cyanobacterial bloom periods (data not shown). In addition to the overall increase in cell density, the annual dynamics of cyanobacteria abundance were characterized by an upward trend during spring, a peak period in summer and autumn and a decline in winter. Moreover, from 2012 to 2014, there were two peaks of cyanobacteria abundance, concentrated in July and October (Figure 2).

### 2.2. Temporal Variation and Spatial Distribution of MCs in Lake Taihu

#### 2.2.1. Monthly Variations of Concentrations of MCs

**Figure 3:**
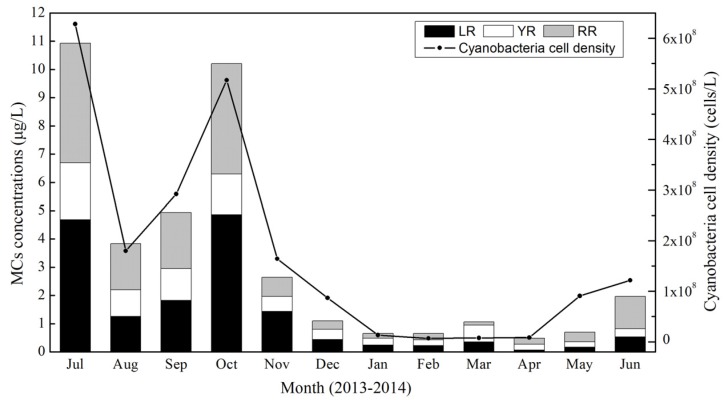
Monthly variability of the concentrations of MCs and cyanobacteria cell density in Lake Taihu.

Three MC variants and cyanobacteria cell density showed similar temporal patterns, with the highest values occurring in July and October, a decline in August and September and relatively low values from January through May (Figure 3).

Three MC variants were detected in all samples during the study period; the mean concentrations of MC-LR, -RR and -YR were 1.28 μg/L, 1.25 μg/L and 0.68 μg/L, respectively, over the course of the entire year. MC-LR and MC-RR accounted for 40% and 39% of total concentrations of MCs, and they varied in a relatively constant fashion from July 2013 to June 2014. MC-YR was usually detected in lower concentrations than MC-LR and MC-RR, comprising 21% of total MCs. The concentration of MC-YR was highest among the three MC variants in March, although absolute concentrations were relatively low during the month (Figure 3).

Total concentrations of MCs, as well as the concentrations of three variants, were significantly higher in July and October than other months. The total concentration of MCs reached a maximum value of 10.93 μg/L in July and the second highest value of 10.21 μg/L in October. Similarly, the highest concentrations of MC-LR were observed in July (4.69 μg/L) and October (4.86 μg/L), which are more than four-times the recommended safety limit of 1 μg/L MC-LR for drinking water (World Health Organization and environmental quality standard for surface water GB3838-2002 by the Ministry of Environmental Protection, the People’s Republic of China). The concentration of MC-LR in the northern parts of Lake Taihu remained above 1 μg/L from July to November.

#### 2.2.2. Seasonal Variations of Concentrations of MCs

**Figure 4:**
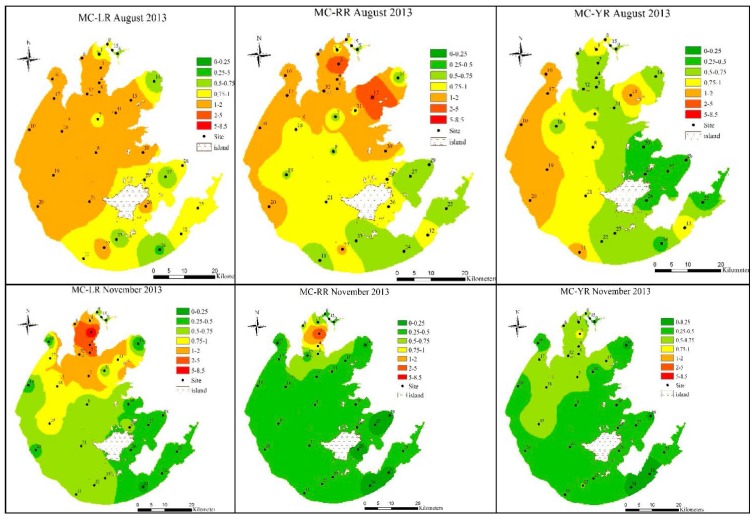

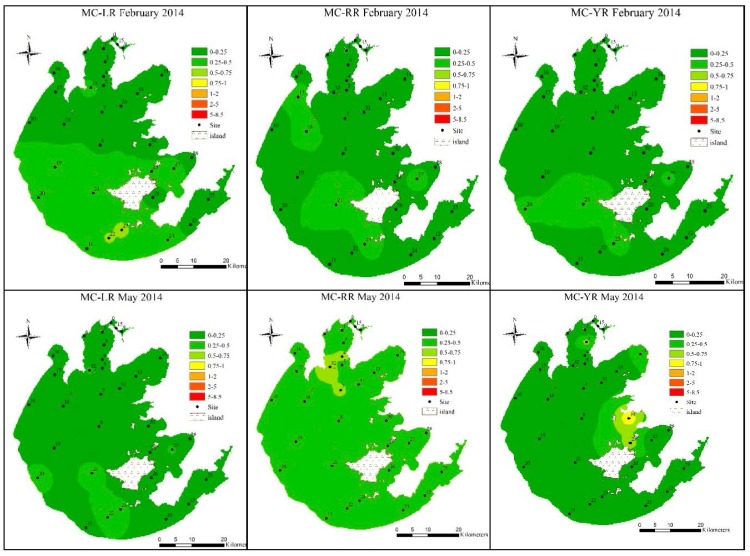
The spatial distribution of microcystins (MCs) in Lake Taihu in August and November 2013 and February and May 2014. The colored areas indicated the concentrations of MC variants in the sampling sites. The color spectrum ranges from green (lowest) to red (highest). The interpolation map was constructed by ArcGIS (Environmental Systems Research Institute Inc., Redlands, CA, USA).

The concentration of MC-LR in August 2013 exceeded 1.0 μg/L in over half of the lake (Figure 4). The maximum MC-LR concentration at this time was 1.91 μg/L, which was detected at Site 17 located in Zhushan Bay. MC-RR was the next most abundant variant and covered about one-third of the lake, with the highest levels at Sites 3 (4.58 μg/L) and 13 (5.17 μg/L). Site 3 is situated in Meiliang Bay, and Site 13 is situated in Gonghu Bay. These three bays have longer hydraulic residence times than other parts of the lake, likely accounting for cyanobacteria bloom formations and high concentrations of MCs. MC-YR concentrations exceeding 1 μg/L accounted for less than one-fourth of the sites, with the highest concentrations concentrated in the west part of Lake Taihu (Figure 4). The highest concentration occurred at Site 10, located in the river estuary named Dapukou, with a value of 1.68 μg/L. Site 10 is close to the port and is susceptible to anthropogenic activities. When the water temperature decreased in November, the MC concentration also significantly declined throughout the entire lake (Figure 4). The concentrations of the three MC variants dropped below the 1.0 μg/L guideline, with the exception of MC-LR and -RR levels in Meiliang Bay, which were 8.18 μg/L and 4.08 μg/L, respectively. The intracellular concentrations of MC-YR ranged from 0 to 0.84 μg/L, with the highest level occurring at Site 3, similar to MC-LR and -RR. In February and May 2014, all three MC variant concentrations were below the safety guideline throughout the entire lake.

### 2.3. Phytoplankton Composition

**Figure 5:**
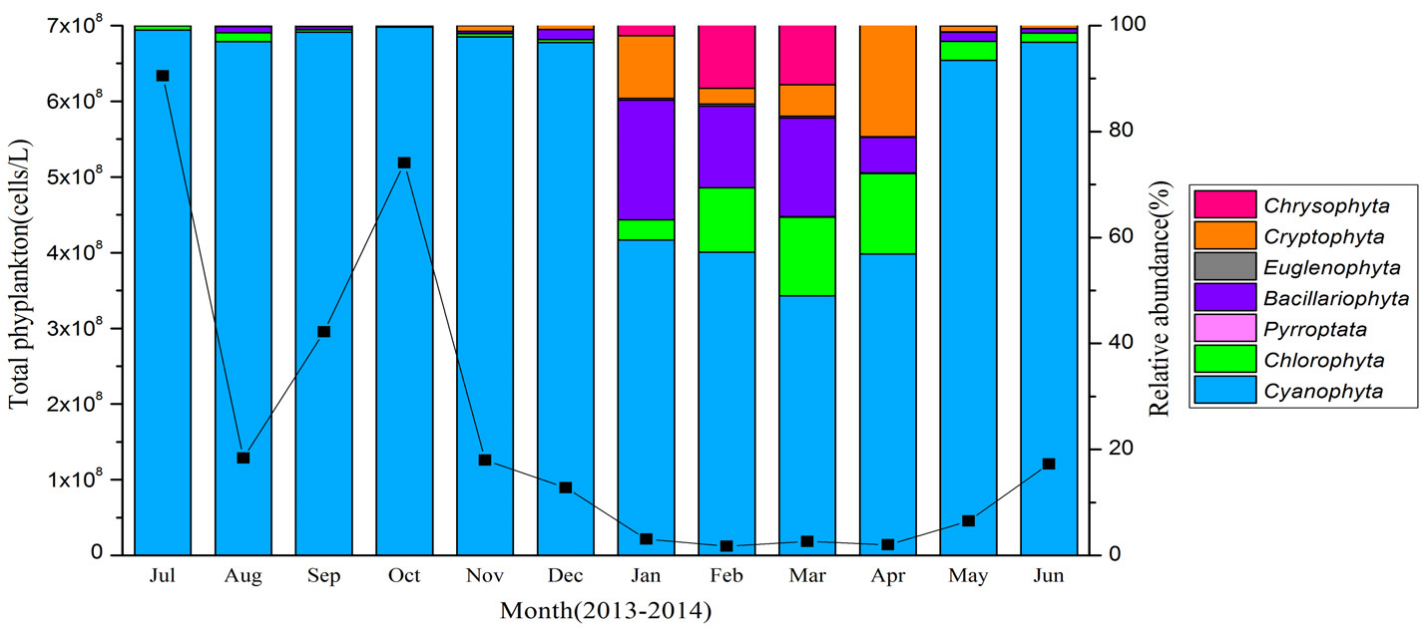
Seasonal variation of the relative abundance of phytoplankton and total phytoplankton density during the study period in Lake Taihu. The solid line represents total phytoplankton density.

Maximum total phytoplankton density was reached in July 2013 with a value of 6.34 × 10^8^ cells/L, dropped sharply in August and then peaked again in October (Figure 5), similar to the patterns exhibited by concentrations of MCs and cyanobacteria cell density (Figure 3). The phytoplankton community was dominated by *Cyanophyta* during summer and autumn seasons, accounting for more than 96% of the total phytoplankton abundance during the study period. In winter, the relative abundance of phytoplankton composition was dominated by *Cyanophyta*, *Bacillariophyta* and *Chlorophyta*. *Bacillariophyta* were relatively more abundant in January, February and March than other months, accounting for 23%, 15% and 19%, respectively, of the total phytoplankton. From February to April, *Chlorophyta* increased its relative abundance from 12% to 15% of the total community. *Cryptophyta* species accounted for 12% and 20% of community relative abundance in January and April, respectively. All other algal divisions accounted for less than 2% of total relative abundance during the study period (Figure 5).

### 2.4. Physicochemical and Biological Parameters

**Figure 6:**
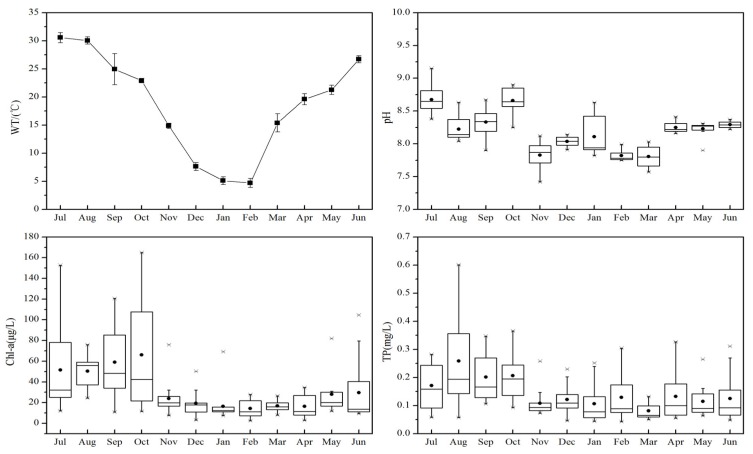
Variation in water temperature (WT), pH, Chl-a and total phosphorus (TP) from July 2013 to June 2014 in Lake Taihu. Error bars (WT) represent the standard deviation. Boxes represent the 25th to 75th percentiles; straight lines within the boxes mark the median; and the small dots indicate the mean. Whiskers below and above the boxes indicate the 10th and 90th percentiles, respectively.

Water temperature (WT) varied from 3.6 °C to 32.03 °C, with the lowest temperature recorded in February; in contrast, the highest temperature was recorded in July (Figure 6). pH ranged from 7.42 to 9.15, with an average of 8.18; the highest values were recorded in July and October, when cell densities were highest. From November to March, pH remained less than 8.20, and from April to June, there was a slight increase ranging from 8.23 to 8.29. Chlorophyll a (Chl-a) concentration ranged from 1.70 to 165.10 μg/L, with the highest values from July to October and a mean annual value of 27.68 μg/L (Figure 6). The averages of total phosphorus (TP) from July to October remained higher than 0.15 mg/L.

All of the water quality parameters measured in this study showed considerable variation on an annual basis (Table 1). Annual mean values revealed generally similar amounts of nitrate and ammonium, with phosphate (PO_4_^3−^–P) accounting for approximately 15% of the TP on an annual basis (Table 1). Chl-a values were particularly variable, with a two-order of magnitude change from minimum to maximum values (Table 1). The mean of the total phytoplankton biomass was 3.28 × 10^3^ mg/L, and the highest value was measured in July and the lowest value in March.

**Table 1:** Mean and range values of physicochemical and biological parameters during the period from July 2013 to June 2014 in Lake Taihu. WT: water temperature; DO: dissolved oxygen; TN: total nitrogen; DTN: total dissolved nitrogen; NH_4_^+^–N: ammonia nitrogen; NO_3_^−^–N: nitrate, NO_2_^−^–N: nitrite; DTP: total dissolved phosphorus; DIC: dissolved inorganic carbon; DOC: dissolved organic carbon.

Variables	Min	Max	Mean ± SD
WT (°C)	3.60	32.00	18.33 ± 9.06
pH	7.42	9.15	8.18 ± 0.28
DO (mg/L)	4.05	14.28	8.99 ± 1.89
Conductivity	220.00	800.00	499.56 ± 115.31
TN (mg/L)	0.48	8.83	2.57 ± 1.49
DTN (mg/L)	0.35	8.46	1.85 ± 1.49
NO_3_^−^–N (mg/L)	0.05	2.96	0.62 ± 0.60
NO_2_^−^–N (mg/L)	0.00	0.49	0.03 ± 0.06
NH_4_^+^–N (mg/L)	0.07	4.48	0.57 ± 0.61
TP (mg/L)	0.02	0.60	0.13 ± 0.09
DTP (mg/L)	0.01	0.31	0.04 ± 0.04
PO_4_^3−^–P (mg/L)	0.00	0.22	0.02 ± 0.03
DIC (mg/L)	13.38	32.01	21.24 ± 4.18
DOC (mg/L)	0.62	8.06	4.27 ± 1.46
Chl-a (μg/L)	1.70	165.10	27.68 ± 27.61
Phytoplankton biomass (mg/L)	0.56	36,681.48	3277.71 ± 5914.13

### 2.5. The Relationship between MC Production and Environmental Parameters

The three MC variants were highly correlated with each other (*r* = 0.649–0.823, *p* < 0.01; Table 2) Cyanobacteria cell density showed a strong positive correlation (*r* > 0.5) with Chl-a, temperature and DOC, but a strong negative correlation with DIC. Total MCs were positively correlated with cyanobacteria, Chl-a, DOC and temperature, but negatively correlated with DIC and all forms of nitrogen, except ammonium (Table 2). MC-LR and MC-YR also showed a positive correlation with cyanobacteria, Chl-a and DOC and a negative relationship with DIC. All of the MC variants were positively related to water temperature and negatively related to DO and nitrate/nitrite (Table 2).

**Table 2:** Spearman correlation coefficients (*r*) for correlations between environmental factors, cyanobacteria and MC concentrations (*n* = 240).

Variable	Cyanobacteria	MCs	MC Variants		
			MC-LR	MC-RR	MC-YR
Cyanobacteria	1	0.678 **	0.659 **	0.705 **	0.586 **
MCs	-	1	0.925 **	0.856 **	0.912 **
MC-LR	-	-	1	0.806 **	0.823 **
MC-RR	-	-	-	1	0.649 **
MC-YR	-	-	-	-	1
WT	0.521 **	0.503 **	0.414 **	0.637 **	0.427 **
pH	0.335 **	0.280 **	0.240 **	0.471 **	0.236 **
DO	−0.390 **	−0.426 **	−0.398 **	−0.585 **	−0.276 **
Conductivity	0.237 **	0.083	−0.017	0.234 **	0.015
TN	−0.101	−0.171 **	−0.224 **	−0.169 **	−0.114
DTN	−0.395 **	−0.429 **	−0.473 **	−0.426 **	−0.360 **
NO_3_^−^–N	−0.451 **	−0.473 **	−0.530 **	−0.475 **	−0.400 **
NO_2_^−^–N	−0.166 *	−0.230 **	−0.236 **	−0.241 **	−0.160 *
NH_4_^+^–N	−0.027	−0.042	0.011	−0.101	−0.033
TP	0.445 **	0.441 **	0.437 **	0.457 **	0.436 **
DTP	0.236 **	0.329 **	0.300 **	0.284 **	0.374 **
PO_4_^3−^–P	0.406 **	0.486 **	0.499 **	0.481 **	0.434 **
DIC	−0.591 **	−0.607 **	−0.657 **	−0.581 **	−0.545 **
DOC	0.537 **	0.568 **	0.606 **	0.548 **	0.543 **
Chl-a	0.743 **	0.617 **	0.547 **	0.618 **	0.611 **

** Correlation is significant at the 0.01 level (2-tailed); * Correlation is significant at the 0.05 level (2-tailed).

**Figure 7:**
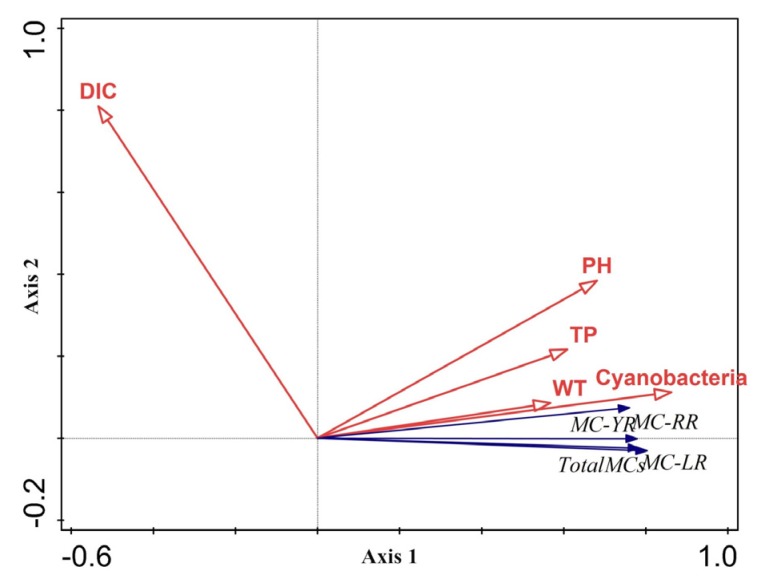
Redundancy analysis biplot displaying the concentrations of intracellular MCs in relation to the key environmental variables in Lake Taihu. Axis 1 explained 61.6% of the variation, and Axis 2 explained 0.2% of the variation.

The RDA illustrated that cyanobacteria accounted for the greatest amount of variation in total MC concentrations (45.8%, *p* = 0.002; Figure 7). Water temperature, TP, pH and DIC also were significant predictors, accounting for 6.9% (*p* = 0.002), 4.0% (*p* = 0.002), 1.7% (*p* = 0.002) and 3.5% (*p* = 0.002), respectively.

## 3. Discussion

Our study suggested that cyanobacteria cell density in Lake Taihu has exhibited a marked increase both in the northern areas and throughout the entire lake over the past ten years. July and October appear to be the two months when cyanobacteria proliferate in Lake Taihu. Comparing our data between the periods of 2005 to 2011 and 2011 to 2014 revealed that the occurrence and dominance of cyanobacterial blooms were more frequent and extensive in the more recent period.

Cyanobacteria accounted for >96% of the total phytoplankton density from May to December, providing a favorable foundation for the production of MC. Summer months, with increasing temperatures, are historically observed when MC production is most abundant [15,32,39,40,41], but in the present study, we measured high concentrations of MCs also in October. This pattern suggests that remediation actions geared to MCs must focus beyond summer and into autumn months, as well.

Lake Taihu is dominated by various macrophytes in its eastern part, whereas the northern part is mainly covered with intense cyanobacteria much of the year. We attempted to evaluate this variation by clustering 14 sites in the northern region and monitoring changes in MC variants on a monthly basis. This was complemented by the placement of 32 sites throughout the entire lake, which were monitored seasonally. The highest concentration of MC variants occurred in the northern lake areas, including Meiliang Bay, Gonghu Bay and Zhushan Bay, the lake center and Dapukou in the west. Fortunately, MC concentrations in the drinking water intake for Wuxi city were below the WHO guideline in our samples, avoiding the problems that faced Lake Erie (USA) [42]. Hydraulic retention time is shorter in this area because of inflows from the Wangyu River, but high concentrations of MCs at adjacent sites present a potential threat for drinking water safety. Water retention time is longer in bays [43], and it is difficult for pollutants to advect or degrade, so water quality is generally worse according to previous reports [32]. The Dapukou area also has high MC values, suggesting that the river flowing into Lake Taihu contains toxins; hence, it is important to monitor the water quality and toxin concentrations in the rivers entering the lake, as well as in-lake sites. With the wind blowing from the southeast in August, the northern part of Lake Taihu may be influenced by cyanobacteria from the lake’s southeast. This, in turn, may result in the eastern portion of the lake becoming relatively clean and safe. Under current conditions, Lake Taihu is losing its ecosystem services and its original function as an important natural resource. For example, it is most likely unsafe to consume aquatic animals harvested in its heavily-polluted regions due to the accumulation of MCs [44,45]. Given the serious contamination with MCs, there is an urgency to limit pollutants from entering Lake Taihu, as well as to explore new techniques to remove MCs that are already present in the lake, such as sediment absorption [46] and biodegradation [47].

Intracellular MC production is influenced directly by MC-containing cyanobacteria and indirectly by environmental factors [25]. Various studies [12,48,49,50] have investigated the role of environmental factors, including temperature, pH, light [51], nitrogen [24] and phosphorus [8] in the regulation of MC production. However, due to geographic distance, differing lake conditions and variation in analytical methods, no consistent conclusions have been reached. In the present study, cyanobacteria density, pH, TP and water temperature were found to be the key variables influencing the variation of MC variants.

Higher temperatures not only promote the dominance of cyanobacteria, but also favor the production of MCs and result in an increase in their concentration [52]. The average temperature in October was 22.9 °C, which is within the optimal range for the growth of cyanobacteria [53]. This positive relationship between temperature and MC production has been observed in Lake Chaohu in China [54], a reservoir in Vietnam [21] and Gonghu Bay [37], suggesting the higher water temperatures in summer and autumn accounted, at least in part, for the higher concentrations of MCs in Lake Taihu. These results have global implications, as they suggest climate warming will lead to more toxic blooms [4]. Among the three MC variants, MC-RR was most related to water temperature (*r* = 0.637). In warm seasons, MC-LR and MC-RR were found to be the dominant variants, but during cold months, MC-YR became dominant; hence, lower temperature may restrict the production of MC-LR and -RR, either directly or indirectly via changes in cyanobacterial taxonomic structure [24]. The mechanisms driving the relationship between water temperature and the production of these three variants need further examination.

In the present study, pH also showed a positive correlation with the production of the three MC variants, which is consistent with the findings in a subtropical reservoir in Singapore [55]. High pH values were observed in July and October when concentrations of MCs were highest, with the average pH above 8.5. A laboratory experiment with *Microcystis aeruginosa* showed that MC production started when pH exceeded 8.4, indicating a lack of free CO_2_ [56]. pH values are usually higher during blooms [57,58] due to the photosynthetic uptake of CO_2_. In turn, a higher pH provides a competitive advantage for many cyanobacteria, because of their strong carbon-concentrating abilities compared to eukaryotic phytoplankton species [57]. It is possible that pH levels favored certain *Microcystis* spp., resulting in greater production of MC-RR compared to MC-LR and -YR. The three MC variants are identical except at the first variable amino acid position, which is occupied by leucine (L), arginine (R) or tyrosine (Y); differences in pH, as well as temperature, may affect their respective biosynthesis, accounting for their spatial and temporal variation. DIC was found to be negatively correlated with each of MC variants, suggesting that increasing DIC concentration may be helpful in controlling the production of MCs. The carbon-nutrient balance hypothesis suggests that a combination of rising CO_2_ and nitrogen enrichment will affect the microcystin composition of harmful cyanobacteria. In nitrogen-rich waters, elevated CO_2_ will induce light-limited conditions, shifting MC composition in cells to higher concentrations of MC-RR, a nitrogen-rich variant [59]. However, as noted above, intensive cyanobacteria growth can remove CO_2_ from the surface water by strong photosynthetic activity, resulting in higher pH and carbon-limited conditions. Thus, concentrations of MCs and the ratios of the three MC variants may be regulated by DIC or CO_2_ availability [60]. In addition, we observed a strong negative correlation between dissolved nitrogen and the production of both total MCs and the variants, suggesting that the role of N warrants more attention. The correlation between TP and intracellular concentration of MCs has been shown to differ among lakes, with positive [14,61], negative or no clear correlation being observed [62]. In the present study, the strongly positive relationship between TP and concentrations of MCs can be attributed to the P concentration’s promotion of both high growth rates of toxic *Microcystis* cells and MC synthesis; this also was observed in a two-year field study in four diverse lake systems across the northeastern U.S. [27]. In our study, Spearman’s correlation coefficients between the three MC variants and TP were similar, suggesting that the composition profile of MC-LR, -RR and -YR was relatively constant regardless of TP concentration. The results were similar to a laboratory experiment with *Microcystis viridis* [63] and a field survey in Bear Lake, Michigan [64]. Cyanobacteria cell density and intracellular MCs were significantly correlated with TP in our study, suggesting the importance of TP in the proliferation of cyanobacterial blooms and MC production. Previously, MC-RR was reported as the dominant toxin form in Lake Taihu [35]. However, our results indicated that both MC-LR and -RR were more abundant than MC-YR. MC-YR was the least abundant variant (<1%) both in Los Padres Lake [16] and in Poyang Lake [41]. However, in this study, MC-YR was a substantial component of total MCs in Lake Taihu. Our results indicated that cyanobacteria biovolume, Chl-a and DOC had a positive effect, and nitrate, nitrite and DIC had a negative effect on MC-YR production. While some studies have focused on MC-LR and MC-RR [48,49,50,61,65], there has been much less focus on MC-YR. Given that MC-YR is almost as toxic as MC-LR according to toxicity equivalent factors (TEF) [66,67], more attention is warranted to better understand how environmental factors influence its production.

An annual study [68] (November 2004 to October 2005) in Lake Taihu found that intracellular MC concentrations reached a peak in July (about 3 μg/L); Wang *et al.* [37] indicated that the peak concentrations of MC were three- to four-times higher in 2008 than in 2005, while Sakai *et al.* [38] detected a maximum MC concentration of 44 μg/L in Meiliang Bay in the summer of 2010. However, these studies investigated only the total MC concentrations in the lake, not those of the variants. Variant type is a very important consideration in a bloom, because each congener has different degrees of toxicity [69]. Results from our study indicate that concentrations of MCs in July were very high, MC-LR was widely distributed through the lake in August and the highest MC-LR concentration (119.77 μg/L) was measured in October. This is the highest concentration of MC-LR ever reported in Lake Taihu, suggesting that the problems of severe cyanobacterial blooms and cyanotoxin contamination in Lake Taihu are getting worse.

In our study, intra-MCs were positively correlated with TP, which was consistent with studies in the USA. [15] and in Lake Taihu [37]. Kotak *et al.* [61] also indicated an effect of TP on MC concentration mediated by the stimulatory effect of TP on *Microcystis* biomass. However, the exact mechanism(s) by which phosphorus influences toxicity is unclear; it may be through changes in cyanobacterial community structure, the type of MC variants that is produced or a combination of the two. While our study clearly points to the role of phosphorus in cyanotoxin production, we do believe it is prudent to control nitrogen, as well, which can play an important role in cyanobacterial blooms [70,71]. Our study suggests that there is a need to better understand phosphorus cycling within Lake Taihu and supports the recommendation to reduce phosphorus inputs to lakes, especially when the goal is to prevent the dominance of toxic cyanobacterial communities.

## 4. Experimental Section

### 4.1. Study Area and Sampling Methods

Lake Taihu (30°56′–31°34′ N, 119°54′–120°36′ E), the third largest freshwater lake in China, is located in the lower Yangtze River Delta in the east of China, with a water surface area of 2338 km^2^, an average depth of 1.9 m, a maximum depth of about 2.6 m and a maximum width of 56 km. It is dominated by a subtropical monsoon climate and has a catchment area of 36,500 km^2^. There are six large cities surrounding the lake with approximately 35 million people using it as a drinking water source [43]. Due to rapid economic development and the intensive use of water resources, water pollution is increasing and water quality is deteriorating rapidly. Coupled with considerable eutrophication and global warming, large-scale cyanobacteria blooms often have occurred during warm seasons in recent years [72]. In order to help understand the variations in cyanobacterial abundance in Lake Taihu, we analyzed long-term datasets of cyanobacteria cell density sampled using two different approaches over the past ten years. The first approach involved quarterly sampling (*i.e.*, February, May, August and November) at 32 sites; the second approach involved monthly sampling at 14 sites that were concentrated mostly in the lake’s northern parts (Figure 1).

Monthly water samples were collected from 14 stations between July 2013 and June 2014, and 32 stations were sampled seasonally in August and November 2013, as well as in February and May 2014. All water samples were collected from the surface layer (0–0.5 m depth) with a Plexiglas water sampler. Five liters of water from each site were preserved in acid-washed, dark plastic bottles pre-conditioned with lake water and were stored immediately in a portable refrigerator (around 4 °C), then transported to the laboratory. The intracellular toxins were extracted from cyanobacterial cells filtered from 1 L lake water on glass-microfiber filters (GF/C, 47 mm × 0.7 µm, Whatman, Brentford, UK). The filters were kept frozen at −40 °C until MC analysis.

### 4.2. Environmental Variables

Water temperature, DO, pH and conductivity were measured *in situ* using a multi-parameter water quality sonde (YSI 6600 V2, Yellow Spring Instruments, Yellow Springs, OH, USA). Chemical analysis of inorganic nutrients in water samples, including TN, TDN, NH_4_^+^–N, NO_3_^−^–N, NO_2_^−^–N, TP, TDP and PO_4_^3−^–P, were determined according to the Chinese national standards for water quality and the methods by APHA (1989). Chl-a concentrations were analyzed with spectrophotometric measurements after extraction in 90% ethanol, set in a hot water bath (85 °C) [73]. DIC and DOC were measured on filtrates filtered through pre-ashed GF/F glass fiber filter (0.8 μm, Whatman, Brentford, UK) and measured with a TOC analyzer.

### 4.3. Phytoplankton

For phytoplankton composition and biomass analyses, a 1-L water sample was fixed with Lugol’s iodine solution at a final concentration of 1% and settled for 48 h prior to microscopic counting. Then, an aliquot of 50 mL was transferred to a plastic bottle for analysis. For each concentrated sample, three slides (each equal to 0.1 mL) were counted as replicates. Phytoplankton species were identified and counted according to Hu *et al.* [74]. More than 100 individuals for each slide were counted under an Olympus microscope at 400× magnification. Total phytoplankton biomass was estimated from cell numbers and cell size measurements, assuming a specific density of 1 g/cm^3^.

### 4.4. MCs Extraction and Analysis

Concentrations of MCs were analyzed according to the solid phase extraction (SPE) method previously reported [75] with minor modification. MCs were extracted by using HLB cartridges and measured by high performance liquid chromatography (HPLC). To determine the concentrations of MCs in the cells, the freeze-dried GF/C filter was extracted with 5% (*v*/*v*) acetic acid using ultra-sonication for 5 min, and the suspension was centrifuged at 10,000 r/min (15 min at 4 °C). This operation was repeated three times, and the supernatants were collected for the next step. The HLB cartridges (200 mg, Oasis^®^, Waters, Milford, MA, USA) were previously activated with methanol (5 mL) and washed with distilled water (5 mL). Afterward, the supernatant was applied at a flow rate of 1 mL/min, with a further washing step with 5% (*v*/*v*) methanol, and a final elution with methanol was performed. A volume of 15 mL was used for the washing step and 10 mL for the elution step described above. Finally, the eluent was dried under N_2_ gas at 40 °C prior to reconstitution in 1.0 mL of methanol. A 500-μL sample was prepared for HPLC analysis.

Quantification of MCs was performed on an Agilent 1200 series HPLC system with a DAD detector (Agilent, Palo Alto, CA, USA) equipped with an ODS column (Agilent Eclipse XDB-C18, 5 μm, 4.6 mm × 150 mm) according to the method described in the literature [75]. The thermostat column is set at 25 °C and a diode-array detector at 238 nm. The mobile phase proportion was at a flow rate of 1 mL/min with the following elution program: linear gradient elution step with 30%–40% B, 70%–60% D for 15 min and then B:D = 30:70 for 5 min. Solvent B is acetonitrile, and Solvent D is distilled water containing 0.05% (*v*/*v*) trifluoroacetic acid. The injection volume is 20 μL. The presence of three main MCs was verified by their UV spectrum and their retention time and quantified using a seven-point calibration curve. Standards for MCs were obtained from Sigma-Aldrich (München, Germany).

### 4.5. Statistical Analysis

All statistical analyses were conducted using SPSS Version 20.0 statistical software (SPSS Inc., Chicago, IL, USA). All of the environmental factors (WT, pH, DO, conductivity, TN, NO_3_^−^–N, NO_2_^−^–N, NH_4_^+^–N, TP, PO_4_^3−^–P, DOC, DIC and Chl-a) were included in the Spearman correlation analysis with cyanobacteria cell density and MC variant concentrations. Redundancy analysis (RDA) was performed to identify the key environmental variables affecting the seasonal change of intracellular MC variants using the multivariate statistical analysis software CANOCO 5.0 (Microcomputer Power, Ithaca, NY, USA). Before the RDA, all environmental variables were log_10_(*x* + 1) transformed to meet the normality and homogeneity of variance. Forward selection of explanatory variables used the partial Monte Carlo permutation test to assess the usefulness of each potential predictor. All considered variables (*N* = 15) explained collectively 68.4% of the total variation, and the five selected variables accounted for approximately 90.6% of the total variability explained by all of the explanatory variables.

## 5. Conclusions

(1) A long-term analysis of cyanobacteria cell density over the past ten years in Lake Taihu revealed that cyanobacteria abundance has shown a distinct increase in its heavily-polluted northern parts and a slight increase throughout the entire lake.
(2) Based on current investigations of 32 sampling sites in Lake Taihu, MC-LR and -RR were the most abundant variants, followed by MC-YR. These three MC variants reached their highest concentrations in July and October.
(3) Redundancy analysis revealed that total phosphorus is a key driving force in the production of MC variants in Lake Taihu. Regulation of phosphorus loads to Lake Taihu is recommended to decrease the threat of cyanobacterial blooms and MCs to human and animal health.
